# Rurality, Health Care Resource Use, and Care Trajectories in Patients With Head and Neck Cancer

**DOI:** 10.1001/jamanetworkopen.2025.4675

**Published:** 2025-04-14

**Authors:** Abigail Thomas, Joseph Clyde Dort, Steven C. Nakoneshny, Thomas Wayne Matthews, Shamir Chandarana, Robert Hart, Khara M. Sauro

**Affiliations:** 1 Department of Community Health Sciences and O’Brien Institute of Public Health, Cumming School of Medicine, University of Calgary, Calgary, Alberta, Canada; 2 Department of Oncology, Arnie Charbonneau Cancer Institute, Cumming School of Medicine, Univerity of Calgary, Calgary, Alberta, Canada; 3 Department of Surgery, Cumming School of Medicine, University of Calgary, Calgary, Alberta, Canada

## Abstract

**Importance:**

Head and neck cancer (HNC) epidemiology varies geographically, and rural populations (typically less affluent) may face barriers accessing cancer care, which could lead to worse outcomes.

**Objective:**

To compare health care resource use between patients with HNC living in urban and rural areas.

**Design, Setting, and Participants:**

Retrospective cohort study in Alberta, Canada. Participants were adult patients (aged ≥18 years) diagnosed with HNC with at least 1 year of posttreatment follow-up between January 2012 and September 2020. Data were analyzed from August to September 2024.

**Exposure:**

Rural location of residence at HNC diagnosis, defined using the forward sortation area of the patients’ postal code.

**Main Outcomes and Measures:**

The primary outcomes were health care resource use and patient care trajectories, including hospital length of stay, 30-day hospital readmissions, emergency department visits, time from diagnosis to first treatment, first type of practitioner seen after hospital discharge, and most common practitioner-to-practitioner transitions.

**Results:**

The cohort included 2189 patients with a median (IQR) age of 63 (55-71) years, who were mostly men (1557 patients [71.1%]) with stage IV cancer (1149 patients [52.5%]) of the tongue (640 patients [29.2%]), of which 375 (17.1%) lived in a rural area. There was no difference in cumulative hospital length of stay between urban and rural patients; however, male patients living in rural areas had a longer surgical hospital length of stay than male patients living in urban areas (incidence rate ratio, 1.24; 95% CI, 1.03-1.50). Patients living in rural areas had more 30-day hospital readmissions (63 patients [16.8%] vs 183 patients [10.1%]) and emergency department visits (median [IQR], 8 [3-17] vs 4 [2-9]) than their urban counterparts. Time to first treatment was longer in patients living in rural areas compared with patients living in urban areas (median [IQR], 64 [46-95] days vs 57 [40-84] days). Patients living in rural areas without comorbid conditions had greater odds of being discharged directly to the care of a general practitioner after a hospital stay than urban patients (odds ratio, 1.97; 95% CI, 1.23-3.15).

**Conclusions and Relevance:**

In this cohort study of patients with HNC, living in rural areas was associated with higher health care resource use than patients living in urban areas. Recommendations specific to patients with HNC living in rural areas may be warranted given these differences.

## Key Points

**Question:**

Does health care resource use or care trajectory differ between patients with head and neck cancer living in urban and rural areas?

**Findings:**

In this cohort study of 2189 patients with head and neck cancer in Alberta, Canada, health care resource use was significantly greater in patients living in rural regions at the time of diagnosis. Care trajectories differed between patients with head and neck cancer living in urban and rural areas.

**Meaning:**

These findings suggest that patients with head and neck cancer living in rural areas have higher health care resource use and different care trajectories than urban patients, indicating that guidelines tailored to the needs of patients diagnosed with head and neck cancer living in rural areas is warranted.

## Teaser

This cohort study evaluates differences in health care resource use between patients with head and neck cancer living in urban and rural areas in Canada.

## Introduction

Head and neck cancers (HNCs) are the sixth most common type of cancer worldwide, but the epidemiology varies geographically.^1^ Variation in the epidemiology of HNC may be related to differences in risk factors such as tobacco use, betel nut use, alcohol use, and exposure to environmental pollutants as well as differences in treatment and surveillance.^1,2^ For example, rural populations have higher rates of tobacco and alcohol use and more frequent exposures to environmental pollutants such as pesticides.^3,4^ Additionally, rural populations tend to be older and less affluent, which are associated with poorer outcomes among patients with HNC.^5,6^ Taken together, people living in rural areas may be at a higher risk of developing HNC and worse outcomes than people living in urban areas.

Furthermore, rural populations can be geographically isolated and may lack health care practitioners (particularly specialists) in their home communities, making it challenging to access needed health care services.^6,7^ Complex illnesses that rely on specialist care, such as HNC, can exacerbate these difficulties as specialist care is typically centralized to urban centers, necessitating travel over greater distances to access care for rural patients.^5,7^ Both patient- and system-level factors associated with living in rural areas affect how patients engage with health care systems. Continuity of care, defined as continuous care by the same practitioner, can be compromised among people living in rural communities, potentially leading to worse outcomes, patient dissatisfaction, and unnecessary health care resource use.^8,9^ Despite differences in risk factors for HNC and possible challenges accessing high-quality cancer care among patients with HNC, little is known about differences in health care resource use, care trajectories, and outcomes between rural and urban patients with HNC.

The objective of this study is to compare health care resource use and care trajectories in patients living in urban and rural areas at the time of their HNC diagnosis. Two distinct research questions were asked to fulfill this overarching objective^1^: are there differences in health care resource use or patient outcomes between patients diagnosed with HNC living in urban and rural locations,^2^ and are there differences in the care trajectory or continuity of care in patients with HNC living in urban and rural locations?

## Methods

### Study Design and Ethics Approval

This retrospective cohort study is reported according to the Strengthening the Reporting of Observational Studies in Epidemiology (STROBE) reporting guideline and Reporting of Studies Conducted Using Observational Routinely Collected Data (RECORD) statement. Ethics approval and a waiver of consent were obtained from the Health Research Ethics Board of Alberta—Cancer Committee.

### Study Setting and Cohort

This study took place in Alberta, Canada; a Western Canadian province with the third largest population in the country (population 4.7 million). In Alberta, health care services are provided by a single health organization, Alberta Health Services (AHS), which is publicly funded and universal. Cancer care for patients with HNC is centralized to 2 tertiary cancer centers located in Calgary and Edmonton, with patients generally cared for in the center closest to their residence.

The Alberta Cancer Registry (ACR) was used to identify a population-based cohort that included all adult patients (18 years or older) who were diagnosed with HNC between January 2012 and September 2020. A diagnosis of HNC was determined by a physician, pathologically confirmed, and included all cancers of the head and neck; patients with thyroid and skin cancer were excluded. Patients under the age of 18 years with HNC were excluded from analysis as the organization of oncology care and disease processes are distinct between adult and pediatric patient population. Patients with less than 1 year of follow-up data after treatment were excluded.

### Data Sources

The ACR contains information on all new cancer diagnoses and deaths in Alberta^10^ and was deterministically linked to the Discharge Abstract Database (DAD), National Ambulatory Care Reporting System (NACRS), eCritical TRACER, and Physician Claims using unique patient identifiers (provincial health care numbers that follow patients for their entire life). The DAD contains administrative, diagnostic, and procedural data on all hospital discharges, with up to 25 *International Statistical Classification of Diseases and Related Health Problems, Tenth Revision (ICD-10)* codes listed for each patient.^11^ NACRS contains data on hospital and community-based ambulatory care such as day surgical procedures, outpatient clinic visits, and emergency department visits.^12^ eCritical TRACER is a population-based bedside clinical information system and repository for all admissions to an intensive care unit within Alberta.^13^ Physician Claims contains administrative data on physician billings for publicly insured health care services in Alberta.

### Variables

Patient demographic and clinical characteristics (eg, age, sex, socioeconomic status, Charlson Comorbidity Index, cancer site, multiple tumors, cancer staging, and primary treatment) were extracted from the ACR and DAD. Quintiles of social and material deprivation from the Pampalon index were used as measures of socioeconomic status.^14^

Geographic location of residence (exposure) was determined using the first 3 digits of the postal code for each patient’s primary residence at the time of their HNC diagnosis, as recorded in the ACR. The first 3 digits of a Canadian postal code are called the forward sortation area and represent well-defined, stable, geographic areas. The second digit of the forward sortation area identifies the postal code as being classified as urban or rural (eg, rural postal codes have a 0 as their second digit).^15^ Using this exposure classification system, patients were classified as rural or urban. The primary outcomes of interest were health care resource use, care trajectories, and continuity of care.

#### Health Care Resource Use

Health care resource included the following 4 variables. The first variable was postoperative hospital length of stay (integer count), or the length of hospital stay associated with a surgery for head and neck cancer. The second was cumulative hospital length of stay. or the number of days spent in hospital over the study period (integer count). The third variable was 30-day all-cause hospital readmission (dichotomized as any vs none). Finally, the fourth variable was emergency department visits in the year following the index hospital discharge (integer count). All measures of health care resource use were abstracted from DAD and NACRS.

#### Care Trajectories

The time from diagnosis (pathology confirmed cancer) to first treatment (integer days, count), first type of practitioner seen after surgical hospital stay (dichotomized as general practitioner [GP] vs specialist), and the most common practitioner-to-practitioner transitions (eg, GP to GP, GP to otolaryngologist, and GP to any nononcology or otolaryngology specialist) were abstracted from Physician Claims. Additional care trajectories and transitions in care (eg, hospital admissions in the 30 days before and after diagnosis [dichotomized as any vs none], and hospital admissions in the 60 days before and after diagnosis [dichotomized as any vs none]) were examined in eTable 1 in Supplement 1.

#### Continuity of Care

Continuity of care was calculated using the modified continuity index (MMCI) and the sequential continuity of care index (SECON) to assess continuity of care separately for GPs and specialist practitioners, such as oncologists, according to the method used by Chen and colleagues^8^ in their paper examining continuity of care in breast cancer survivors.^16^ The continuity indices are proportions (range, 0-1) where lower values indicate less continuity and higher values indicate greater continuity. We did not differentiate between different practitioners with the same specialty, as unique practitioner identifiers were not available.

Secondary outcomes included patient outcomes, such as complications during the surgical hospital admission and overall hospital mortality during the study period.^17^ Both were extracted from the DAD and dichotomized as present or absent (*ICD-10* codes used to identify complications of interest are provided in eTable 2 in Supplement 1).

### Statistical Analysis

Descriptive statistics (ie, means, SDs, counts, and percentages) were used to describe the cohort. Two-sided *t* tests with equal variance were used to examine differences in continuous demographic characteristics and outcomes of interest between patients with HNC from urban and rural communities, while χ^2^ tests were used for categorical variables. Medians, IQRs, and nonparametric tests were used to describe and examine variables with skewed distributions. When the assumptions of the χ^2^ test were not met, Fisher exact test was used as an alternative.

Multivariable logistic regression was used to evaluate the association between first type of practitioner seen after discharge from surgical hospital admission, 30-day hospital readmissions, hospital mortality, and complications experienced while in hospital and location of residence. Multivariable negative binomial regression was used to evaluate the association between surgical hospital length of stay, ED visits, and total number of physician claims and location of residence.

A top-down approach, moving from the most complex model to the minimum set of adjustments, was used for all statistical modeling. Age (continuous), sex (dichotomous), social deprivation (referring to relations within family, workplace, and community, divided into quintiles with 1 indicating the least deprived individuals and 5 denoting the most deprived individuals), material deprivation (referring to deprivation of everyday modern goods and conveniences, broken into quintiles with 1 indicating the least deprived individuals and 5 denoting the most deprived individuals), cancer stage at diagnosis, and Charlson Comorbidity Index (categorical [no comorbid conditions, 1 comorbid condition, 2 or more comorbid conditions]) were assessed as effect modifiers using 1-way interaction terms in regression models before considering confounding. Interaction terms with a *P* value less than .05 were considered effect modifiers, while confounding was determined using clinical judgement. Categorical variables found to be effect modifiers (eg, quintile of material deprivation) had their categories collapsed, if appropriate, to ensure a clear association was presented.

The data were explored for missingness before analysis. For analyses where there were missing data, a case-wise deletion approach was used. An α of .05 was selected a priori for all analyses, and a Bonferroni correction was applied separately to Tables 1 and 2 to account for multiple comparisons, which changed the set α to .013 (Table 1) and .003 (Table 2).^18^ All analyses were conducted in Stata 17.0 (StataCorp).^19^

**Table 1.** Demographic Characteristics of the Study Cohort Stratified by Geographic Location of Primary Residence

Characteristic	Participants, No (%)			*P* value^a^
	Cohort (N = 2189)	Rural (n = 375)	Urban (n = 1814)	
Sex				
Male	1557 (71.1)	272 (72.5)	1285 (70.8)	.51
Female	632 (28.9)	103 (27.5)	529 (29.2)	
Age at diagnosis, median (IQR), y	63 (55-71)	64 (57-73)	62 (55-71)	.003
Health care zone				
Calgary	1420 (64.9)	30 (8.0)	1390 (76.6)	<.001
Central	286 (13.1)	1 (0.3)	285 (15.7)	
Edmonton	483 (22.0)	344 (91.7)	139 (7.7)	
Charlson Comorbidity Index				
0	896 (40.9)	147 (39.1)	749 (41.3)	.72
1	106 (4.8)	18 (4.8)	88 (4.9)	
≥2	1188 (54.3)	211 (56.1)	977 (53.9)	
Cancer staging				
0	18 (0.8)	4 (1.1)	14 (0.8)	.90
I	281 (12.8)	47 (12.5)	234 (12.9)	
II	175 (8.0)	28 (7.5)	147 (8.1)	
III	223 (10.2)	36 (9.6)	187 (10.3)	
IV	1149 (52.5)	206 (54.9)	943 (52.0)	
Missing	343 (15.7)	54 (14.4)	289 (15.9)	
Cancer site				
Tongue	640 (29.2)	104 (27.8)	536 (29.5)	NA
Larynx	321 (14.7)	68 (18.2)	253 (14.0)	
Tonsil	313 (14.3)	59 (15.7)	254 (14.0)	
Parotid gland	165 (7.5)	27 (7.2)	138 (7.6)	
Floor of mouth	117 (5.3)	21 (5.6)	96 (5.3)	
Nasopharynx	106 (4.8)	7 (1.9)	99 (5.5)	
Oropharynx	89 (4.1)	9 (2.4)	80 (4.4)	
Gum	86 (3.9)	10 (2.7)	76 (4.2)	
Palate	64 (2.9)	13 (3.5)	51 (2.8)	
Mouth, other and unspecified	62 (2.8)	7 (1.9)	55 (3.0)	
Hypopharynx	59 (2.7)	12 (3.2)	47 (2.6)	
Pyriform sinus	60 (2.7)	17 (4.5)	43 (2.4)	
Lip	35 (1.6)	11 (2.9)	24 (1.3)	
Major salivary glands, other and unspecified	30 (1.4)	6 (1.6)	24 (1.3)	
Accessory sinuses	18 (0.8)	1 (0.3)	17 (0.9)	
Lip, oral cavity, and pharynx, other and unspecified	15 (0.7)	2 (0.5)	13 (0.7)	
Nasal cavity and middle ear	9 (0.4)	1 (0.3)	8 (0.4)	
Primary treatment				
Chemotherapy	100 (4.6)	9 (2.4)	91 (5.0)	.23
Immunotherapy	38 (1.7)	5 (1.3)	33 (1.8)	
Radiotherapy	564 (25.8)	102 (27.2)	462 (25.5)	
Surgery	1288 (58.8)	225 (60.0)	1063 (58.6)	
Observation	14 (0.6)	4 (1.1)	10 (0.6)	
None, refused, or unknown	185 (8.5)	30 (8.0)	155 (8.6)	
No. of tumors				
1	1997 (91.2)	339 (90.4)	1658 (91.4)	.53
≥2	192 (8.8)	36 (9.6)	156 (8.6)	
Quintile of material deprivation^b^				
1, Least deprivation	345 (17.7)	31 (9.9)	314 (19.2)	<.001
2	322 (16.5)	41 (13.1)	281 (17.1)	
3	472 (24.2)	117 (37.5)	355 (21.7)	
4	344 (17.6)	45 (14.4)	299 (18.2)	
5, Most deprivation	469 (24.0)	78 (25.0)	391 (23.8)	
Quintile of social deprivation^b^				
1, Least deprivation	251 (12.9)	0	251 (15.3)	<.001
2	320 (16.4)	106 (34.0)	214 (13.1)	
3	448 (23.0)	72 (23.1)	376 (22.9)	
4	425 (21.8)	93 (29.8)	332 (20.2)	
5, Most deprivation	508 (26.0)	41 (13.1)	467 (28.5)	

Abbreviation: NA, not applicable.

^a^ Bonferroni correction applied to account for multiple comparisons; as a result, an α of .013 determines statistical significance for Table 1.

^b^ Data only presented for 1951 patients.

**Table 2.** Outcomes, Stratified by Geographic Location of Primary Residence

Outcome	Participants, No. (%)			*P* value^a^
	Cohort (N = 2189)	Rural (n = 375)	Urban (n = 1814)	
Health care resource use				
Length of surgical hospital stay, median (IQR), d^b^	8 (2-15)	7 (2-16)	8 (2-15)	.47
Total days spent in hospital over the entire study period, median (IQR)	15 (6-37)	16 (6-45)	15 (6-36)	.08
30-d Hospital readmissions^b^	246 (11.2)	63 (16.8)	183 (10.1)	<.001
Total No. of ED visits, median (IQR)	5 (2-9)	8 (3-17)	4 (2-9)	<.001
Admitted to an ED 90 d prediagnosis	151 (6.9)	22 (5.9)	129 (7.1)	.39
Admitted to an ED 90 d postdiagnosis^c^	50 (2.3)	4 (1.1)	46 (2.5)	.08
Admitted to an ED 90 d presurgery^b^	70 (5.4)	13 (5.8)	57 (5.3)	.77
Total No. of physician claims after diagnosis, median (IQR)	125 (79-191)	125 (75-194)	125 (79-191)	.84
Patient outcomes				
Died in hospital during the study period	367 (16.8)	83 (22.1)	284 (15.7)	.002
Experienced an AE during surgical hospital admission^b^	255 (19.6)	54 (24.0)	201 (18.7)	.07
Experienced an in-hospital AE	592 (27.0)	99 (26.4)	493 (27.2)	.76
Continuity of care indices				
MMCI, mean (SD)^d^	0.99 (0.03)	0.99 (0.03)	0.99 (0.02)	.46
SECON, mean (SD)^d^	0.66 (0.12)	0.68 (0.12)	0.65 (0.11)	<.001
Transitions in care				
First type of practitioner seen after discharge from surgical hospital admission^b^				.06
GP	229 (24.7)	46 (30.9)	183 (23.6)	
Specialist	697 (75.3)	103 (69.1)	594 (76.5)	
GP to GP	728 (33.3)	158 (42.1)	570 (31.4)	<.001
GP to other	372 (17.0)	46 (12.3)	326 (18.0)	.01
GP to OTOL	90 (4.1)	12 (3.2)	78 (4.2)	.33

Abbreviations: AE, adverse event; ED, emergency department; GP, general practitioner; MMCI, Modified Continuity Index; OTOL, otolaryngologist; SECON, Sequential Continuity of Care Index.

^a^ Bonferroni correction applied to account for multiple comparisons; as a result, an α of .003 determines statistical significance for Table 2.

^b^ For parameters conditional on a patient having undergone surgery as a form of treatment (eg, surgical hospital length of stay), the sample size for the cohort was 1301.

^c^ When assumptions of the χ^2^ test were violated, Fisher exact test was used as an alternative.

^d^ The MMCI and SECON were only able to be calculated for 1861 patients (328 patients were missing data in at least 1 of the variables used to calculate these measures).

## Results

### Demographic Characteristics

A total of 2189 patients diagnosed with HNC in Alberta with at least 1 year of follow-up data were included. Detailed demographic characteristics of this cohort are presented in Table 1. The median (IQR) age at diagnosis was 63 (55-71) years, 1557 (71.1%) were male, and (54%) had 2 or more comorbid conditions (Table 1). The most common cancer site was the tongue (640 patients [29.2%]), and most patients were diagnosed with stage IV cancer (1149 patients [52.5%]). Patients had a mean (SD) of 1.3 (0.3) treatment modalities with the primary modality being surgery (1288 patients [58.8%]) followed by radiotherapy (564 patients [25.8%]).

At the time of their cancer diagnosis, 375 patients (17.1%) lived in rural areas (Table 1); these patients were older (64 years vs 62 years) and more fell into the lowest quintile of material deprivation compared with urban patients (78 patients [25.0%] vs 391 patients [23.8%]). In contrast, a greater proportion of urban patients fell into the lowest quintile of social deprivation compared with rural patients (urban: 467 patients [28.5%] vs rural: 41 patients [13.1%]). There were no statistical differences in sex, cancer staging, or primary treatment between patients living in urban and rural locations at the time of their HNC diagnosis (Table 1).

### Health Care Resource Use

#### Cumulative Hospital Length of Stay

There was no demonstrated difference in hospital length of stay between patients with HNC living in urban and rural areas. (Table 2). The median (IQR) for patients in urban areas was 15 (6-36) days and was 16 (6-45) days for patients in rural areas.

#### Surgical Stay

**Figure 1.**
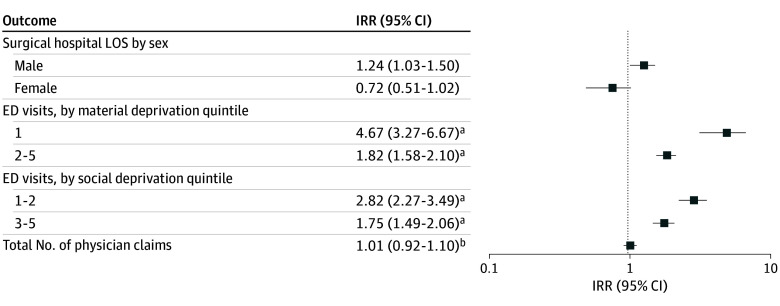
Adjusted Incidence Rate Ratios (IRRs) for Measures of Health Care Resources Use As quintile of material and social deprivation were either confounders or modifiers of the association(s) of interest, the sample size was limited to patients with complete data for these 2 variables (1951, as 238 patients were missing data on material and social deprivation). Lower quintiles indicate less deprivation. ED indicates emergency department; LOS, length of stay. ^a^Adjustments made for age at diagnosis and comorbidities. ^b^Adjustments made for age at diagnosis, comorbidities, quintile of material deprivation, stage at diagnosis, and quintile of social deprivation.

Unadjusted analysis of surgical hospital length of stay was unable to detect differences between urban and rural patients with HNC (median [IQR], 8 [2-15] days vs 7 [2-16] days). This association was modified by patients’ biological sex, demonstrating no association between location of residence in female patients (IRR, 0.72; 95% CI, 0.51-1.02) and demonstrating that the length of stay was longer for male patients living in rural communities than male patients living in urban communities after adjusting for age at diagnosis, comorbidities, cancer stage at diagnosis, and quintile of social and material deprivation (IRR, 1.24; 95% CI, 1.03-1.50) (Figure 1; eTable 3 in Supplement 1).

#### Readmission

**Figure 2.**
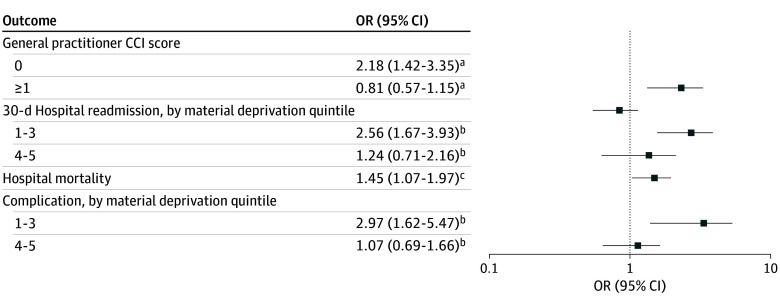
Adjusted Odds Ratios (ORs) of Measures of Health Care Resource Use As quintile of material and social deprivation were either confounders or modifiers of the association(s) of interest, the sample size was limited to patients with complete data for these 2 variables (1951, as 238 patients were missing data on material and social deprivation). Lower quintiles indicate less deprivation. CCI indicates Charlson Comorbidity Index. ^a^Adjustments made for age at diagnosis, sex, stage at diagnosis, and quintiles of material and social deprivation. ^b^Adjustments made for age at diagnosis, comorbidities, and quintile of social deprivation. ^c^Adjustments for age at diagnosis, comorbidities, quintile of social deprivation, and quintile of material deprivation

Rural patients with HNC had more frequent 30-day hospital readmissions than their urban counterparts (63 patients [16.8%] vs 183 patients [10.1%]). The association between 30-day hospital readmissions and location of residence was modified by quintile of material deprivation, such that patients with lower material deprivation living in rural areas (those in the upper 3 quintiles) had greater odds of 30-day hospital readmissions (OR, 2.56; 95% CI, 1.67-3.93) adjusting for age at diagnosis, comorbidities, and quintile of social deprivation. There was no demonstrated difference in the odds of 30-day hospital readmissions between patients living in urban and rural locations who had the lowest material deprivation (those in the fourth and fifth quintiles), adjusting for age at diagnosis, comorbidities, and quintile of social deprivation (OR, 1.12; 95% CI, 0.62-2.03) (Figure 2). None of the other covariates modified or confounded the association between readmission and location of residence (eTable 4 in Supplement 1).

#### ED Visits

Patients with HNC living in rural areas visited the ED more frequently than urban patients (median [IQR], 8 [3-17] vs 4 [2-9]) (Table 2). Patients diagnosed with HNC living in rural regions visited the ED more than their urban counterparts, even with modification by both quintile of material and quintile of social deprivation and adjustments for confounding by age at diagnosis and comorbidities. The magnitude of association was greater in the uppermost quintile of material deprivation (IRR, 4.67; 95% CI, 3.27-6.67) than the other 4 quintiles of material deprivation (IRR, 1.82; 95% CI, 1.58-2.10) (Figure 1). The magnitude of association was also greater in the upper 2 quintiles of social deprivation (IRR, 2.82; 95% CI, 2.27-3.49), compared with the lower 3 quintiles (IRR, 1.75; 95% CI, 1.49-2.06) (Figure 1). None of the other covariates modified or confounded the association between ED visits and location of residence (eTable 5 in Supplement 1).

### Care Trajectory and Care Continuity

#### Referrals After Hospital Discharge

Patients with HNC living in a rural location were more frequently referred directly to the care of a GP after their surgical hospital admission than urban patients (30.9% vs 23.6%); however, this difference was not statistically significant (Table 2). Patients with HNC living in rural regions without any comorbid conditions had greater odds of being discharged directly to a GP after hospital discharge (OR, 1.97; 95% CI, 1.23-3.15), though this association did not persist in patients with comorbid conditions (OR, 0.81; 95% CI, 0.56-1.17) with adjustments for age at diagnosis, cancer stage at diagnosis, and quintiles of material and social deprivation (Figure 2; eTable 6 in Supplement 1).

#### Practitioner-to-Practitioner Transitions

Patients with HNC living in rural regions waited longer for first treatment than urban patients (median [IQR], 64 [46-95] days vs 57 [40-84] days). Patients with HNC from rural communities were transferred between GPs more than urban patients (158 patients [42.1%] vs 570 patients [31.4%]), while urban patients were transferred between GPs and other practitioner types more frequently than rural patients (326 patients [18.0%] vs 46 patients [12.3%]). The frequency of transfer between GPs and otolaryngologists was not statistically different between patients with HNC living in urban and rural regions (Table 2).

#### Continuity of Care Indices

Using the MMCI to assess care continuity, no difference was observed between patients with HNC from urban and rural regions. However, using the SECON to assess care continuity, patients diagnosed with HNC from rural regions had better care continuity than urban patients (mean [SD], 0.68 [0.12] vs 0.65 [0.11]) (Table 2).

### Patient Outcomes

#### Hospital Mortality

In-hospital mortality was higher in rural patients than urban patients (83 patients [22.1%] vs 284 patients [15.7%]). This remained true after accounting for comorbidities, age at diagnosis, cancer stage at diagnosis, and quintile of material and social deprivation using logistic regression (OR, 1.46; 95% CI, 1.06-2.02) (Figure 2; eTable 7 in Supplement 1).

#### Complications

Unadjusted analysis was unable to find differences in the occurrence of complications during surgical hospital admissions between patients with HNC living in urban and rural areas (261 patients [14.4%] vs 42 patients [11.1%]). However, after accounting for potential confounders and effect modifiers, patients with HNC from rural areas who fell into the first or second quintile of material deprivation had greater odds of experiencing a complication than patients with HNC from urban locations, adjusting for age at diagnosis, comorbidities, and quintile of social deprivation (OR, 3.41; 95% CI, 1.82-6.38) (Figure 2). This association did not persist in the third through fifth quintiles of material deprivation, adjusting for age at diagnosis, comorbidities, and quintile of social deprivation. None of the other covariates modified or confounded the association between complications and location of residence (eTable 8 in Supplement 1).

## Discussion

Patients with HNC from rural areas were older and lower on the social and material deprivation scales than their urban counterparts, except in the lowest quintile of social deprivation, where a larger proportion of patients with HNC were from urban regions. This may reflect that individuals with precarious housing tend to live in urban regions. Health care resource use differed between urban and rural patients with HNC, with those living in rural areas having higher acute care use after their index hospital discharge, more frequent ED visits (regardless of their social or material deprivation indices), and higher rates of 30-day hospital readmissions (association only present in patients who fell into the upper 3 quintiles of material deprivation) than urban patients. Differences in care trajectories between urban and rural patients with HNC were apparent and included differences in time to first treatment, hospital discharge destinations, and the type of practitioners seen after hospital discharge.

The literature exploring rurality and socioeconomic status in patients with HNC is heterogeneous, largely due to differences in definitions for these 2 interrelated concepts. As area-based measures of socioeconomic status are traditionally used, the question of whether rurality and socioeconomic status are collinear also remains unanswered. In the present study, we found that rurality and socioeconomic status had independent effects on patient outcomes; however, given the varied definitions of rurality and socioeconomic status, the findings in this study that rurality and socioeconomic status are not collinear may not hold true for other measures and definitions. The present study chose the Pampalon Index, which offers a comprehensive measure of area-based deprivation specific to the Canadian context and parses out social and material deprivation into separate indices, providing a nuanced perspective on socioeconomic status.^15^ Despite the differences in definitions and indices for socioeconomic status, the classification of rurality in Canada is rather clearly defined by the federal government and takes into consideration distance from services and population density.^16^ Given the heterogeneity in definitions and complex relationship between rurality and socioeconomic status, when we compare our study with the broader body of literature, similarities and differences are both readily apparent.

Living in rural areas and lower socioeconomic status have been associated with worse oncologic outcomes among patients with HNC; however, the effects of rurality and socioeconomic status on outcomes in patients with HNC vary by outcome and patient population. For example, one study in Canadian patients with HNC found no difference in overall survival between urban and rural patients,^20^ while another found that disease-specific survival rates were lower in rural patients.^21^ Yet another study found that socioeconomic status was associated with survival in patients with HNC, with those from low socioeconomic areas having worse overall survival.^22^ Moreover, there appear to be differences on the effects of rurality and socioeconomic status in the American context compared with the Canadian context where tumor stage at diagnosis differed based on rurality.^23^ Some of the differences in findings between these studies may be related to population characteristics but may also reflect differences in health care systems and health services.

Access to care can be challenging for patients with HNC living in rural locations,^24^ which can in turn be associated with worse oncologic outcomes. Indeed, patients with HNC living in rural locations often have to leave their community to receive treatments for HNC, and are less likely to meet treatment timing benchmarks.^25^ Our study did not examine challenges with access to care specifically; however, we found that rural patients had greater odds of seeing a GP directly following a surgical admission than urban patients if they had no comorbid conditions. This pattern of care may indicate disparities in access to appropriate care after discharge for HNC surgery at tertiary care centers. Future research is needed to expand on our findings to innovate care delivery for patients living in rural, remote, and underserved areas which could improve oncologic outcomes and equitable cancer care.

Tailored cancer care for patients with HNC living in rural areas is needed. Updated local practice guidelines^26^ did not account for the centralization of specialist care to urban centers, leaving a gap in the recommendations for care of patients living in rural areas. The findings of the present study, when considered in the context of findings from a scoping review looking at transitions in care among patients with cancer,^27^ suggest a need to focus on transitions in care during treatment for patients living in rural areas. More specifically, the transition from hospital to home is particularly challenging, especially for patients living in rural communities who are typically discharged to GPs rather than specialist care. This was highlighted in our study and another study^28^ that examined the impact of rural residence in critically ill patients; they found that critically ill patients living in rural regions had more frequent discharges to GPs, and fewer visits to specialist practitioners in the year following discharge from ICU. Patients could be better supported through this transition by developing a discharge plan for rural patients that is directly communicated to both the patient and their primary care practitioner to improve documentation of the course in the hospital and recommendations for follow-up care, as well as by increasing access to specialist care in rural areas via telehealth.^29^ Future research should focus on targeted discharge preparation, additional training for GPs, and on the appropriateness of telehealth in rural patients with HNC.

### Strengths and Limitations

A unique feature and strength of our study is that the effects of geography and socioeconomic status on health care resource use and patient outcomes was explored to understand the independent contribution of rurality. Most other studies examining rurality and socioeconomic status do not adequately address the complex relationship between these 2 variables and simply adjust for either socioeconomic status or rurality using statistical modeling.

The primary limitation of our study is the lack of a criterion standard definition of rural vs urban residence. As full postal codes were unavailable, the forward sortation area was used to define urban vs rural residence, which confers a loss of precision to smaller geographical areas. In addition, we defined urban or rural location of residence based on where patients were living at the time of their HNC diagnosis. If patients moved during the study period, such as moving from a rural to an urban location, it was not reflected in a change in exposure status. Also, the demographic characteristics of our cohort and the context in which our study was conducted (eg, a publicly funded health care system, treatment guidelines, and practices) should be taken into consideration when determining the generalizability of the findings to other contexts. Finally, due to the limited number of covariates available in our data, there is a possibility that there is unmeasured confounding contributing to the association between our outcomes and exposure (location of residence).

## Conclusions

In this cohort of patients with HNC, patients living in rural areas had higher acute care resource use than their urban counterparts, regardless of their socioeconomic profile. Because specialist services were centralized to urban centers, it may have impacted the care trajectories for patients with HNC living in rural areas, leading patients to have received care more frequently from GPs. Developing recommendations specific to the care of patients with HNC living in rural areas is warranted, as their socioeconomic profile, health care resource use, and care trajectories differed from urban patients.

## Notes

Supplement 1. **eTable 1.** Care trajectories and transitions in care relative to HNC diagnosis in patients from urban and rural locations **eTable 2.** Categories of complications and their* ICD-10* Codes **eTable 3.** Negative binomial regression model output for covariates included when modeling surgical hospital length of stay **eTable 4. **Logistic regression model output for covariates included when modeling 30-day hospital readmissions, stratified by material deprivation **eTable 5.** Negative binomial regression model output for covariates included when modeling ED visits, stratified by both social and material deprivation **eTable 6.** Logistic regression model output for covariates included when modeling first type of practitioner seen after hospital discharge, stratified by presence or absence of comorbid conditions **eTable 7.** Logistic regression model output for covariates included when modeling hospital mortality **eTable 8. **Logistic regression model output for covariates included when modeling complications

Supplement 2.
Data Sharing Statement

## References

[1] Johnson DE, Burtness B, Leemans CR, Lui VWY, Bauman JE, Grandis JR. Head and neck squamous cell carcinoma. Nat Rev Dis Primers. 2020;6(1):92. doi:10.1038/s41572-020-00224-3 33243986 PMC7944998

[2] International Agency for Research on Cancer. Human cancer: known causes and prevention by organ site. 2024. Accessed December 12, 2023. https://monographs.iarc.who.int/human_cancer_known_causes_and_prevention_organ_site/

[3] Leonel ACL da S, Bonan RF, Pinto MB, Kowalski LP, Perez DE. The pesticides use and the risk for head and neck cancer: a review of case-control studies. Med Oral Patol Oral Cir Bucal. 2021;26(1):e56-e63. doi:10.4317/medoral.23962 32701932 PMC7806356

[4] Zhou X, Su Z, Deng H, Xiang X, Chen H, Hao W. A comparative survey on alcohol and tobacco use in urban and rural populations in the Huaihua District of Hunan Province, China. Alcohol. 2006;39(2):87-96. doi:10.1016/j.alcohol.2006.07.003 17134661

[5] Haggerty JL, Roberge D, Lévesque JF, Gauthier J, Loignon C. An exploration of rural-urban differences in healthcare-seeking trajectories: implications for measures of accessibility. Health Place. 2014;28:92-98. doi:10.1016/j.healthplace.2014.03.005 24793139

[6] Sibley LM, Weiner JP. An evaluation of access to health care services along the rural-urban continuum in Canada. BMC Health Serv Res. 2011;11(1):20. doi:10.1186/1472-6963-11-20 21281470 PMC3045284

[7] Wong ST, Regan S. Patient perspectives on primary health care in rural communities: effects of geography on access, continuity and efficiency. Rural Remote Health. 2009;9(1):1142. doi:10.22605/RRH1142 19298094

[8] Chen YY, Hsieh CI, Chung KP. Continuity of care, follow-up care, and outcomes among breast cancer survivors. Int J Environ Res Public Health. 2019;16(17):3050. doi:10.3390/ijerph16173050 31443512 PMC6747467

[9] Skolarus TA, Zhang Y, Hollenbeck BK. Understanding fragmentation of prostate cancer survivorship care: implications for cost and quality. Cancer. 2012;118(11):2837-2845. doi:10.1002/cncr.26601 22370955 PMC4860006

[10] Alberta Health Services. Alberta cancer registry. Accessed September 21, 2023. https://www.albertahealthservices.ca/cancer/Page17367.aspx

[11] Canadian Institute for Health Information. Discharge abstract database (DAD) metadata. Accessed March 1, 2023. https://www.cihi.ca/en/discharge-abstract-database-metadata-dad

[12] Canadian Institute for Health Information. National ambulatory care reporting system (NACRS) metadata. Accessed March 1, 2023. https://www.cihi.ca/en/national-ambulatory-care-reporting-system-metadata-nacrs

[13] Brundin-Mather R, Soo A, Zuege DJ, . Secondary EMR data for quality improvement and research: a comparison of manual and electronic data collection from an integrated critical care electronic medical record system. J Crit Care. 2018;47:295-301. doi:10.1016/j.jcrc.2018.07.021 30099330

[14] Government of Canada. Chronic diseases in Canada. 2009. Accessed April 18, 2023. https://www.canada.ca/en/public-health/services/reports-publications/health-promotion-chronic-disease-prevention-canada-research-policy-practice/vol-29-no-4-2009/deprivation-health-planning-canada.html

[15] Statistics Canada. Postal code conversion file (PCCF), reference guide [2011]. Accessed Decembr 12, 2023. https://www150.statcan.gc.ca/n1/en/catalogue/92-153-G

[16] Gulliford M, Naithani S, Morgan M. What is ‘continuity of care’? J Health Serv Res Policy. 2006;11(4):248-250. doi:10.1258/135581906778476490 17018200

[17] Southern DA, Burnand B, Droesler SE, . Deriving *ICD-10* codes for patient safety indicators for large-scale surveillance using administrative hospital data. Med Care. 2017;55(3):252-260. doi:10.1097/MLR.0000000000000649 27635599

[18] Bland JM, Altman DG. Multiple significance tests: the Bonferroni method. BMJ. 1995;310(6973):170. doi:10.1136/bmj.310.6973.170 7833759 PMC2548561

[19] Stata. Stata statistical software: release 18. Accessed October 3, 2022. https://www.stata.com/support/faqs/resources/citing-software-documentation-faqs/

[20] Kim JD, Firouzbakht A, Ruan JY, . Urban and rural differences in outcomes of head and neck cancer. Laryngoscope. 2018;128(4):852-858. doi:10.1002/lary.26836 28940575

[21] Zhang H, Dziegielewski PT, Jean Nguyen TT, . The effects of geography on survival in patients with oral cavity squamous cell carcinoma. Oral Oncol. 2015;51(6):578-585. doi:10.1016/j.oraloncology.2015.03.012 25865551

[22] Weizman B, Golan N, Ronen O. Effect of socioeconomic status on survival in patients with head and neck cancer. Head Neck. 2021;43(10):3001-3009. doi:10.1002/hed.26788 34137115

[23] Lawrence LA, Heuermann ML, Javadi P, Sharma A. Socioeconomic status and rurality among patients with head and neck cancer. Otolaryngol Head Neck Surg. 2022;166(6):1028-1037. doi:10.1177/01945998211019278 34126811

[24] Checklin M, O’Halloran R, Foster AM, . The health care experiences of people with head and neck cancer: a scoping review. Head Neck. 2024;46(1):74-85. doi:10.1002/hed.27558 37882242

[25] Foley J, Wishart LR, Ward EC, . Exploring the impact of remoteness on people with head and neck cancer: utilisation of a state-wide dataset. Aust J Rural Health. 2023;31(4):726-743. doi:10.1111/ajr.13005 37280733

[26] Harris JR, Lau H, Surgeoner BV, ; Members of the Alberta Provincial Head and Neck Tumour Team. Health care delivery for head-and-neck cancer patients in Alberta: a practice guideline. Curr Oncol. 2014;21(5):e704-e714. doi:10.3747/co.21.1980 25302041 PMC4189575

[27] Thomas A. Transitions in care and healthcare resource use in critically ill patients living in urban and rural settings: a retrospective cohort study. University of Calgary. 2023. Accessed June 14, 2024. https://hdl.handle.net/1880/117199

[28] Gujral K, Scott JY, Ambady L, . A primary care telehealth pilot program to improve access: associations with patients’ health care utilization and costs. Telemed J E Health. 2022;28(5):643-653. doi:10.1089/tmj.2021.0284 34559017

[29] Teng K, Russo F, Kanuch S, Caron A. Virtual care adoption-challenges and opportunities from the lens of academic primary care practitioners. J Public Health Manag Pract. 2022;28(6):599-602. doi:10.1097/PHH.0000000000001548 36037465 PMC9555588

