# Supplementary material for: Rurality, Health Care Resource Use, and Care Trajectories in Patients With Head and Neck Cancer
Source: JAMA Netw Open. 2025 Apr 14;8(4):e254675. doi: 10.1001/jamanetworkopen.2025.4675 (PMC11997726; doi:10.1001/jamanetworkopen.2025.4675)
Supplement: Supplement 1. — eTable 1. Care trajectories and transitions in care relative to HNC diagnosis in patients from urban and rural locations eTable 2. Categories of complications and their ICD-10 Codes eTable 3. Negative binomial regression model output for covariates included when modeling surgical hospital length of stay eTable 4. Logistic regression model output for covariates included when modeling 30-day hospital readmissions, stratified by material deprivation eTable 5. Negative binomial regression model output for covariates included when modeling ED visits, stratified by both social and material deprivation eTable 6. Logistic regression model output for covariates included when modeling first type of practitioner seen after hospital discharge, stratified by presence or absence of comorbid conditions eTable 7. Logistic regression model output for covariates included when modeling hospital mortality eTable 8. Logistic regression model output for covariates included when modeling complications [file jamanetwopen-e254675-s001.pdf]

Supplemental Online Content

Thomas A, Dort JC, Nakoneshny SC, et al. Rurality,health care resource use, and care trajectories in head and neck cancer patients. *JAMA Netw Open*. 2025;8(4):e254675. doi:10.1001/jamanetworkopen.2025.4675

eTable 1. Care trajectories and transitions in care relative to HNC diagnosis in patients from urban and rural locations

eTable 2. Categories of complications and their *ICD-10* Codes

This supplemental material has been provided by the authors to give readers additional information about their work.

© 2025 Thomas A et al. *JAMA Network Open*.

eTable 1. Care trajectories and transitions in care relative to HNC diagnosis in patients from urban and rural locations

**Outcome**	**Cohort**[N=2,189]	**Rural**[N=375]	**Urban**[N=1,814]	**P-value**
Any hospital admission in the 30-days pre diagnosis, n (%)	207 (9.5)	41 (10.9)	166 (9.2)	0.283
Any hospital admission in the 30-days post diagnosis, n (%)	251 (11.5)	50 (13.3)	201 (11.1)	0.213
Any hospital admission in the 60-days pre diagnosis, n(%)	214 (9.8)	42 (11.2)	172 (9.5)	0.308
Any hospital admission in the 60 days following diagnosis, n (%)	746 (34.1)	126 (33.6)	620 (34.2)	0.830

© 2025 Thomas A et al. *JAMA Network Open*.

eTable 2. Categories of complications and their ICD-10 Codes

From Southern and colleagues 2017 paper, “Deriving ICD-10 Codes for Patient Safety Indicators for Large-scale Surveillance Using Administrative Hospital Data” (18).

**Category**	**ICD-10 Codes**
Hospital-acquired infections	A02.0, A02.1, A04.4, A04.5, A04.7, A04.8, A04.9, A08.0, A08.1, A41.0, A41.1, A41.2, A41.4, A41.50, A41.51, A41.52, A41.58, A41.80, A41.88, A41.9, A49.0, B30.9, B37.3, B37.4, B37.7, B37.80, B37.81, B95.6, B95.7, B95.8, B96.1, B96.2, B96.4, B96.5, B96.81, B96.88, B97.4, G00.3, J15.0, J15.1, J15.2, J15.5, J15.6, J15.9, J18.1, J21.0, J85.3, J86.0, J86.9, J95.01, K65.0, N39.0, N99.51, O75.30, O85.00, O86.00, O86.10, O86.20, O86.30, O86.80, P36.0, P36.1, P36.2, P36.3, P36.4, P36.8, P36.9, P38, R57.2, T81.4, T82.6, T82.7, T83.5, T83.6, T84.53, T84.54, T84.60, T84.61, T84.63, T84.64, T84.65, T84.68, T84.7, T85.7, T87.42, T87.46, T87.47, T87.48
Decubitus ulcer	L89.0, L89.1, L89.2, L89.3, L89.8, L89.9
Endocrine and metabolic complications	E10.10, E10.63, E10.64, E11.0, E11.10, E11.11, E11.63, E11.64, E13.63, E14.63, E15, E16.0, E27.2, E89.1, E89.2, E89.3, G37.2, T50.3
Venous thromboembolic events	I26.0, I26.9, I80.1, I80.2, I82.2, O87.102
Cardiac complications	I20.0, I20.1, I20.88, I20.9, I21.0, I21.1, I21.2, I21.3, I21.4, I21.9, I22.0, I22.1, I22.8, I22.9, I46.1, I46.9, I47.2, I48.1, I49.00, I49.01, I50.0, I50.1, I50.9, J81, O74.20, S26.811, T82.0, T82.1, T82.2, T82.5, T82.6, T82.7, T82.8, T82.9
Respiratory complications	J15.0, J15.1, J15.2, J15.5, J15.6, J15.9, J18.1, J21.0, J38.01, J38.02, J38.09, J69.0, J69.8, J85.3, J86.0, J86.9, J94.2, J95.00, J95.01, J95.02, J95.03, J95.08, J95.1, J95.2, J95.5, J95.80, J95.81, J95.88, J95.9, J96.0, S20.2, S22.200, S22.300, S22.400, S22.410, S22.490, S27.000, S27.001, S27.100, S27.200, S27.300, S27.310, T17.3, T17.4, T17.5, T17.8, T17.9, T71, T79.7, T81.81
Hemorrhagic events	D62, D68.3, J94.2, J95.00, O71.701, O71.704, O71.801, O72.00, O72.10, O72.20, O90.20, P12.0, S06.4, S06.5, S06.6, S27.100, S27.200, S27.300, S36.090, S36.091, S36.150, S36.151, S36.800, S36.810, S37.000, S37.300, T79.2, T81.0
Drug-related adverse events	D68.3, E16.0, E88.3, H91.0, I95.2, O74.50, T36.0, T36.1, T36.5, T36.8, T36.9, T37.8, T38.0, T38.3, T39.0, T39.1, T39.3, T39.8, T40.2, T40.3, T40.4, T40.6, T41.2, T41.3, T42.0, T42.1, T42.4, T42.6, T427, T43.0, T43.2, T43.4, T43.5, T43.8, T44.5, T44.7, T45.0, T45.1, T455, T457, T45.8, T46.0, T46.1, T46.2, T46.4, T46.5, T47.4, T48.0, T48.6, T49.0, T50.1, T50.2, T50.9, T80.8, T80.9, T81.80, T88.2, T88.3, T88.6
Adverse events related to fluid management	E86.0, E86.8, E87.7, G37.2, T50.3, T80.8, T80.9
Obstetrical complications	O08.6, O29.50, O70.20,O70.30, O70.90, O71.10, O71.11, O71.18, O71.30, O71.40, O71.50, O71.60, O71.70, O71.80, O72.00, O72.10, O72.20, O74.20, O74.30, O74.50, O74.60, O74.80, O75.10, O75.30, O75.40, O75.40, O75.60, O85.00, O86.00, O86.10, O86.20, O86.30, O86.80, O87.10, O89.40, O89.50, O89.80, O90.00, O90.10, O90.20 P03.3, P12.0, P12.3, P12.8, P13.4, P14.3, P15.4, P15.8, P36.0, P36.1, P36.2, P36.3, P36.4, P36.8, P36.9, P38
Surgical and anesthesia-related complications	H59.80, M96.6, O75.401, O75.40, O86.00, O90.00, S26.811, S27.001, S36.091, S36.151, S36.411, S36.461, S37.111, S37.211, S37.311, T81.0, T81.1, T81.2, T81.3, T81.52, T81.58, T81.59, T81.6, T81.81, T81.88, T81.9, O29.50, O74.20, O74.30, O74.50, O74.60, O74.80, O89.40, O89.50, O89.80, T41.2, T41.3, T88.2, T88.3, T88.4, T88.5
Traumatic injuries arising in hospital (nonprocedural)	S01.00, S01.01, S01.10, S01.20, S01.30, S01.40, S01.50, S01.70, S01.80, S01.90, S02.000, S02.100, S02.200, S02.300, S02.480, S02.490, S02.5, S02.890, S03.0, S05.0, S05.1, S05.8, S05.9, S06.0, S06.1, S06.25, S06.35, S06.4, S06.5, S06.6, S06.85, S06.9, S09.0, S09.8, S09.9, S10.1, S10.9, S13.48, S14.38, S20.2, S20.4, S20.8, S22.200, S22.300, S22.400, S22.410, S22.490, S27.000, S27.100, S27.200, S27.300, S27.310, S27.810, S27.860, S30.0, S30.1, S30.80, S30.81, S30.88, S30.9, S31.200, S31.400, S32.100, S32.400, S32.500,

© 2025 Thomas A et al. *JAMA Network Open*.

	S32.700, S32.800, S33.5, S35.1, S35.2, S35.5, S36.090, S36.150, S36.460, S36.610, S36.810, S37.000, S37.090, S37.110, S37.190, S37.210, S37.290, S37.300, S37.310, S37.390, S37.610, S39.08, S39.8, S39.9, S40.0, S40.8, S40.9, S41.10, S41.11, S42.010, S42.020, S42.090, S42.190, S42.200, S42.210, S42.220, S42.280, S42.290, S42.300, S42.390, S42.400, S42.480, S43.000, S43.090, S43.100, S46.00, S46.08, S49.7, S49.8, S49.9, S50.0, S50.1, S50.7, S50.8, S50.9, S51.00, S51.01, S51.70, S51.80, S51.90, S52.000, S52.100, S52.300, S52.500, S52.580, S52.590, S52.600, S52.800, S59.8, S59.9, S60.0, S60.2, S60.7, S60.8, S60.9, S61.00, S61.70, S61.80, S61.90, S62.000, S62.500, S62.690, S62.800, S63.100, S63.59, S69.8, S69.9, S70.0, S70.1, S70.8, S70.9, S71.10, S71.11, S72.000, S72.010, S72.080, S72.090, S72.100, S72.190, S72.200, S72.300, S72.410, S72.420, S72.490, S72.800, S72.900, S73.000, S73.090, S74.18, S75.0, S79.9, S80.0, S80.1, S80.7, S80.8, S80.9, S81.00, S81.01, S81.80, S81.81, S81.90, S81.91, S82.000, S82.100, S82.200, S82.300, S82.400, S82.500, S82.600, S82.800, S82.890, S83.6, S89.8, S89.9, S90.0, S90.1, S90.3, S90.7, S90.8, S90.9, S91.00, S91.10, S91.20, S91.30, S92.000, S92.300, S92.400, S92.500, S93.49, S99.8, S99.9, T00.1, T00.8, T00.9, T09.0, T11.0, T11.1, T13.0, T13.1, T14.0, T14.9, T20.0, T20.2, T21.0, T21.1, T21.2, T21.3, T22.0, T22.4, T23.0, T23.2, T24.0, T24.2, T25.0, T25.2, T71, T79.2, T79.6, T79.7
Delirium	F05.0, F05.1, F05.8, F05.9
Central nervous system complications	E11.0, E15, F05.0, F05.1, F05.8, F05.9, G00.3, G37.2, G97.2, O74.30, O89.40, S06.0, S06.1, S06.25, S06.35, S06.4, S06.5, S06.6, S06.85, S06.9
Gastrointestinal complications	A02.0, A04.4, A04.5, A04.7, A04.8, A04.9, A08.0, A08.1, B37.80, B37.81, K22.3, K65.0, K91.0, K91.3, S27.810, S27.860, S36.150, S36.151, S36.411, S36.460, S36.461, S36.610, T18.1, T18.2, T18.3, T18.9, T28.2, T85.5
Severe events	G37.2, I21.0, I21.1, I21.2, I21.3, I21.4, I21.9, I22.0, I22.1, I22.8, I22.9, I26.0, I46.1, I46.9, I47.2, I49.00, I49.01, J96.0, K22.3, K65.0, O74.20, O74.30, O75.10, O75.40, R57.1, R57.2, R57.8, T71, T80.0, T80.5, T81.1, T88.2, T88.3, T88.4, T88.6

© 2025 Thomas A et al. *JAMA Network Open*.

eTable 3: Negative binomial regression model output for covariates included when modelling surgical hospital length of stay.

**Covariate**	**Coefficient (95% CI)**	**Std. error**	**z**	**P > |z|**
**Men**				
Age at diagnosis	0.021 (0.015 – 0.027)	0.0031	6.75	< 0.001
Quintile of material deprivation	0.039 (-0.11 – 0.091)	0.0259	1.53	0.125
Quintile of social deprivation	0.074 (0.018 – 0.128)	0.0281	2.62	0.009
Comorbidities	0.145 (0.069 – 0.220)	0.0385	3.75	< 0.001
Stage at diagnosis	0.252 (0.187 – 0.316)	0.0330	7.64	< 0.001
**Women**				
Age at diagnosis	0.023 (0.016 – 0.031)	0.0039	5.94	< 0.001
Quintile of material deprivation	-0.016 (-0.105 – 0.074)	0.0456	-0.34	0.733
Quintile of social deprivation	0.085 (-0.012 – 0.181)	0.0491	1.72	0.085
Comorbidities	0.132 (0.007 – 0.258)	0.0641	2.07	0.039
Stage at diagnosis	0.103 (0.001 – 0.205)	0.0519	1.98	0.047

© 2025 Thomas A et al. *JAMA Network Open*.

eTable 4: Logistic regression model output for covariates included when modelling 30-day hospital readmissions, stratified by material deprivation.

**Covariate**	**Coefficient (95% CI)**	**Std. error**	**z**	**P > |z|**
**Quintiles 1 through 3 of material deprivation**				
Age at diagnosis	0.033 (0.018 – 0.049)	0.0078	4.24	< 0.001
Comorbidities	0.113 (-0.091 – 0.316)	0.1039	1.08	0.279
Quintile of social deprivation	0.109 (-0.040 – 0.260)	0.0765	1.44	0.151
**Quintiles 4 and 5 of material deprivation**				
Age at diagnosis	0.015 (-0.003 – 0.032)	0.0088	1.65	0.099
Comorbidities	-0.001 (-0.222 – 0.221)	0.1128	-0.00	0.996
Quintile of social deprivation	-0.024 (-0.186 – 0.139)	0.0830	-0.28	0.777

© 2025 Thomas A et al. *JAMA Network Open*.

eTable 5: Negative binomial regression model output for covariates included when modelling ED visits, stratified by both social and material deprivation.

Covariate	Coefficient (95% CI)	Std. error	z	P > |z|
**Quintiles 1 and 2 of social deprivation**				
Age at diagnosis	0.009 (0.002 – 0.016)	0.0034	2.71	0.007
Comorbidities	0.021 (-0.066 – 0.111)	0.0453	0.48	0.629
**Quintiles 3 through 5 of social deprivation**				
Age at diagnosis	-0.001 (-0.006 – 0.004)	0.0024	-0.39	0.698
Comorbidities	0.015 (-0.046 – 0.077)	0.0317	0.50	0.619
**Quintile 1 of material deprivation**				
Age at diagnosis	0.006 (-0.0022 – 0.013)	0.0039	1.40	0.162
Comorbidities	0.075 (-0.035 – 0.185)	0.0561	1.43	0.182
**Quintiles 2 through 5 of material deprivation**				
Age at diagnosis	0.001 (-0.003 – 0.006)	0.0022	0.65	0.517
Comorbidities	0.009 (-0.048 – 0.066)	0.02911	0.31	0.758

© 2025 Thomas A et al. *JAMA Network Open*.

eTable 6: Logistic regression model output for covariates included when modelling first type of provider seen after hospital discharge, stratified by presence or absence of comorbid conditions.

Covariate	Coefficient (95% CI)	Std. error	z	P > |z|
**No comorbid conditions**				
Age at diagnosis	0.003 (-0.010 – 0.017)	0.0068	0.42	0.673
Sex	-0.129 (-0.511 – 0.253)	0.1947	-0.66	0.509
Quintile of material deprivation	-0.041 (-0.177 – 0.095)	0.0694	-0.59	0.553
Quintile of social deprivation	-0.119 (-0.259 – 0.019)	0.0710	-1.68	0.092
Stage at diagnosis	-0.039 (-0.182 – 0.103)	0.0729	-0.54	0.590
**One or more comorbid conditions**				
Age at diagnosis	0.006 (-0.005 – 0.018)	0.0056	1.17	0.242
Sex	0.111 (-0.203 – 0.424)	0.1600	0.69	0.489
Quintile of material deprivation	0.057 (-0.041 – 0.156)	0.0503	1.14	0.255
Quintile of social deprivation	-0.046 (-0.149 – 0.057)	0.0525	-0.88	0.379
Stage at diagnosis	-0.065 (-0.203 – 0.073)	0.0706	-0.92	0.357

© 2025 Thomas A et al. *JAMA Network Open*.

eTable 7: Logistic regression model output for covariates included when modelling hospital mortality.

**Covariate**	**Coefficient (95% CI)**	**Std. error**	**z**	**P > |z|**
Age at diagnosis	0.032 (0.021 – 0.042)	0.0054	5.80	< 0.001
Quintile of material deprivation	0.012 (-0.080 – 0.104)	0.0471	0.25	0.799
Quintile of social deprivation	0.095 (-0.003 – 0.194)	0.0504	1.89	0.059
Comorbidities	0.363 (0.215 – 0.511)	0.0756	4.80	< 0.001
Stage at diagnosis	0.521 (0.363 – 0.679)	0.0805	6.47	< 0.001

© 2025 Thomas A et al. *JAMA Network Open*.

eTable 8: Logistic regression model output for covariates included when modelling complications.

Covariate	Coefficient (95% CI)	Std. error	z	P > |z|
**Quintiles 1 and 2 of material deprivation**				
Age at diagnosis	0.003 (-0.014 – 0.020)	0.0088	0.33	0.738
Comorbidities	-0.091 (-0.334 – 0.151)	0.1238	-0.74	0.461
Quintile of social deprivation	-0.065 (-0.245 – 0.114)	0.0915	-0.71	0.476
**Quintiles 3 through 5 of material deprivation**				
Age at diagnosis	-0.003 (-0.016 – 0.011)	0.0071	-0.37	0.711
Comorbidities	0.041 (-0.140 – 0.222)	0.0924	0.44	0.658
Quintile of social deprivation	0.003 (-0.124 – 0.129)	0.0645	0.05	0.964

© 2025 Thomas A et al. *JAMA Network Open*.
