# Supplementary material for: Rurality, Health Care Resource Use, and Care Trajectories in Patients With Head and Neck Cancer
Source: JAMA Netw Open. 2025 Apr 14;8(4):e254675. doi: 10.1001/jamanetworkopen.2025.4675 (PMC11997726; doi:10.1001/jamanetworkopen.2025.4675)
Supplement: Supplement 2. — Data Sharing Statement [file jamanetwopen-e254675-s002.pdf]

Data Sharing Statement

Thomas. Rurality, Health Care Resource Use, and Care Trajectories in Patients With Head and Neck Cancer. *JAMA Netw Open*. Published April 14, 2025.
doi:10.1001/jamanetworkopen.2025.4675

Data
Data available: Yes
Data types: Deidentified participant data
How to access data: Data will be made available upon reasonable request to the corresponding author kmsauro@ucalgary.ca
When available: beginning date: 01-01-2025

Supporting Documents
Document types: Statistical/analytic code
How to access documents: Analysis code will be made available upon reasonable request to the corresponding author kmsauro@ucalgary.ca
When available: With publication

Additional Information
Who can access the data: Anyone making a reasonable request to the corresponding author.
Types of analyses: for any purpose
Mechanisms of data availability: After approval of a proposal
